# 91756 A participatory approach to develop regional health priorities for clinical and translational research

**DOI:** 10.1017/cts.2021.749

**Published:** 2021-03-30

**Authors:** Shinobu Watanabe-Galloway, Paul Estabrooks, David Palm, Sean Navarrette, Heidi Keeler, Keyonna King, Emily Frankel

**Affiliations:** University of Nebraska Medical Center - UNMC

## Abstract

ABSTRACT IMPACT: Regional health issues can be best addressed at the population-level and input from the communities is vital for prioritization of health issues. OBJECTIVES/GOALS: The Great Plains IDeA-CTR (GP IDeA-CTR) was developed to increase clinical and translational research (CTR) that can address regional health priorities. Here we describe a collaborative process used to identify regional health priorities using existing surveillance data and community input. METHODS/STUDY POPULATION: We used a participatory approach that included a partnership between the GP IDeA CTR Community-Engagement and Biostatistics, Epidemiology, and Research Design Cores to ensure priorities were data driven and also aligned with community-based perceptions of need. First, aggregated surveillance data across Nebraska, North Dakota, and South Dakota was presented to the GP IDeA CTR Community Advisory Board (CAB). Second, CAB members formed small groups and considered the information and generated priority health area lists. Third, small group lists were considered and discussed by the full CAB to finalize priority areas. Finally, the CAB reviewed the priorities annually thereafter. RESULTS/ANTICIPATED RESULTS: We identified priority areas for CTR that included (1) behavioral health, (2) injury prevention, (3) obesity, (4) technology to improve health care access, (5) connecting clinical/community services, and (5) addressing health disparities. These priorities align with population-based surveillance data that show lack of mental health care access, high prevalence of obesity, higher incidence of accidents, and existing racial, ethnic, and geographic health disparities. The CAB highlighted that research was also needed to improve how people can access the health innovations developed through CTR to address the other priority health issues with a goal to have an impact on population health. DISCUSSION/SIGNIFICANCE OF FINDINGS: By integrating data- and community-driven approaches we identified regional health priority areas that if addressed, can have significant impact in the GP IDeA CTR region. The priorities are listed on all GP IDeA-CTR funding announcements to encourage CTR in these areas.

